# Isorhynchophylline Mitigates Bone Loss in OVX Mice by Modulating Inflammatory Responses, Oxidative Stress, Gut Microbiota Composition and SCFAs Production

**DOI:** 10.1002/fsn3.70803

**Published:** 2025-08-25

**Authors:** Yitao Lei, Xiao Zhao, Shihui Liang, Yanming Liu, Yilin Zhou, Yingtong Zhang, Shimei Li, Qunwei Dong, Ping Sun

**Affiliations:** ^1^ Department of Endocrinology The First Affiliated Hospital of Guangdong Pharmaceutical University Guangzhou China; ^2^ Guangzhou University of Chinese Medicine Guangzhou China; ^3^ Huanghuagang Street Community Health Service Center Guangzhou China

**Keywords:** anti‐inflammatory, gut microbiota, isorhynchophylline, osteoporosis, ovariectomized mice, SCFAs

## Abstract

Osteoporosis (OP) is a systemic skeletal disorder characterized by reduced bone mass and deteriorated bone architecture. Isorhynchophylline (IRN), an active alkaloid derived from the plant *Uncaria rhynchophylla*, is known for its significant anti‐inflammatory and antioxidant properties. However, its potential role in mitigating bone loss has not been investigated. This study examines the protective effects of IRN against bone loss in ovariectomized (OVX) mice, with a focus on its regulation of inflammatory responses, oxidative stress, gut microbiota composition, the production of short‐chain fatty acids (SCFAs) and the intestinal barrier. The research methods include network pharmacology, molecular docking, and in vivo experiment. Network pharmacology analysis identified 50 intersecting targets between IRN and OP, with molecular docking demonstrating strong binding affinities of IRN to EGFR and GSK3B. GO and KEGG enrichment analyses revealed that IRN is involved in many key pathways related to cell survival, inflammation, and oxidative stress, such as the PI3K‐Akt signaling pathway. In vivo assessment showed that IRN treatment reduced serum levels of the inflammatory cytokines, such as TNF‐α, IL‐1β and IL‐6, while increasing serum levels of the anti‐inflammatory cytokine IL‐10 in OVX mice. IRN effectively managed the oxidative stress in the OVX model, characterized by lowered serum levels of NO, iNOS, and ROS. Combination of 16S rRNA sequencing and GC–MS analysis found that IRN modulated the gut microbiota composition, reducing the abundance of *Campylobacterota* and *Helicobacter*, and increasing the level of butanoic acid. Additionally, immunohistochemical analysis indicates that IRN repaired the compromised intestinal barrier in OVX mice by upregulating the expression of tight junction proteins, including ZO‐1, Claudin‐1, and Occludin. These results suggest that IRN, through its anti‐inflammatory and antioxidant properties, modulation of gut microbiota composition, enhancement of SCFAs production, and protection of the intestinal barrier, provides a new therapeutic approach for natural products to alleviate bone loss.

## Graphical abstract

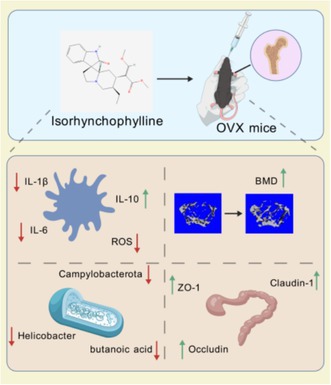

## 1 Introduction

Osteoporosis (OP) is a metabolic bone disease characterized by reduced bone mass and deteriorated bone microarchitecture, increasing fracture risk and posing a significant global public health challenge (Pouresmaeili et al. 2018). Postmenopausal osteoporosis (PMOP) is the most common OP form in women, primarily driven by estrogen decline after menopause. Estrogen deficiency promotes osteoclast activation and bone resorption, leading to decreased bone volume and accumulation of bone microdamages (Li, Chen, Lu, and Yu 2020; Wu et al. 2021). The first‐line oral anti‐osteoporosis drugs include bisphosphonates, selective estrogen receptor regulators, and calcitriol, but long‐term use of these therapies can cause atypical femoral fractures, thrombosis, and peripheral edema (Wang, Qu, et al. 2024; Skjødt et al. 2019). Therefore, there is an urgent need to develop novel therapies as alternative options.

Accumulating evidence has shown that bioactive compounds extracted from natural Chinese herbs are beneficial to preserve bone microarchitecture through anti‐inflammatory and antioxidant activities without significant safety concerns over a long period of treatment (Liu et al. 2019; Hong et al. 2021; Chen et al. 2019). Isorhynchophylline (IRN) is a naturally occurring tetracyclic oxindole alkaloid extracted from the herb *Uncaria rhynchophylla*. Previous studies have shown the anti‐inflammatory and antioxidant properties of IRN in rheumatoid arthritis, osteoarthritis, and Alzheimer disease (Wu, Bian, et al. 2023; Li et al. 2022; Zeng et al. 2021). Chronic inflammation and oxidative stress are major contributors to osteoporosis pathogenesis, fueling osteoclastic bone resorption and impairing osteoblastic bone formation (Compston et al. 2019; Boyle et al. 2003; Sobacchi et al. 2013). IRN has strong potential to mitigate inflammation and stress oxidation and alleviate osteoporotic symptoms, which remain to be systematically examined.

The gut microbiota, a multifaceted ecosystem, plays a crucial role in maintaining host health. Emerging evidence underscores the significant role it plays in bone metabolism and in the development of osteoporosis (Guo et al. 2023; Li et al. 2024). For instance, studies have shown that postmenopausal women with osteoporosis exhibit distinct gut microbiota profiles compared to those with normal bone density, suggesting that specific microbial communities may influence bone metabolism (Xiao et al. 2024). The gut microbiota not only affects bone health directly by regulating calcium and phosphate absorption, but also modulates inflammatory processes, the secretion of short‐chain fatty acids (SCFAs) and the integrity of the intestinal barrier that are critical in the pathogenesis of osteoporosis (Cheng et al. 2021). SCFAs are gut microbiota‐derived metabolites that enhance osteogenic differentiation of bone marrow stromal cells and improve bone mineral density (Wu, Chen, et al. 2023). They also help to maintain gut barrier integrity, regulate intestinal absorption, modulate immune cells, and promote the secretion of anti‐inflammatory cytokine, thereby potentially mitigating the inflammatory processes associated with osteoporosis (Van Der Hee and Wells 2021; Vinolo et al. 2011; Zhu et al. 2021; Weaver 2015). Compromised intestinal barrier function, often associated with postmenopause, has been shown to increase intestinal permeability, allowing for the translocation of bacterial metabolites into the bloodstream and triggering systemic inflammation that further exacerbates bone loss (Rettedal et al. 2021; Zhang et al. 2024). Given the essentialness of gut microbiota, SCFAs, and the intestinal barrier in bone health, this study aimed to explore the in vivo protective effect of IRN against excessive bone loss and the underlying mechanistic roles of gut microbiota biology.

## 2 Materials and Methods

### 2.1 Network Pharmacology Analysis

The primary active compounds of *Uncaria rhynchophylla* were screened from the TCMSP database (https://tcmspw.com/tcmsp.php) based on criteria of oral bioavailability (OB) ≥ 30% and drug likeness (DL) ≥ 0.18. IRN was identified as the main active monomer for further analysis. The compound's 2D structure was retrieved in SDF format by entering its CAS number in PubChem (https://pubchem.ncbi.nlm.nih.gov). To assess absorption and drug‐likeness, the SMILES format of IRN was submitted to the SwissADME database (https://www.swissadme.ch/), where high GI absorption and two or more positive drug‐likeness indicators confirmed its suitability for study. The SMILES format of IRN was further used in Swiss Target Prediction (STP, https://www.swisstargetprediction.ch/) to predict its core target genes based on a probability threshold. Disease‐related targets for osteoporosis were identified using the GeneCards (https://www.genecards.org), OMIM (https://www.omim.org), and DisGeNET (https://www.disgenet.org) databases, merging the gene lists from these databases. Overlapping targets between the active compound and osteoporosis‐associated genes were visualized using a Venn diagram created by an online analysis tool Venny2.1.0.

To construct protein–protein interactions (PPI), the predicted targets of IRN were uploaded to the STRING database (https://cn.string‐db.org/) with the protein species set to “ *Homo sapiens* ” and a minimum interaction score threshold of 0.4. Unconnected nodes were excluded from the network to enhance clarity, and the resulting interaction data were saved in TSV format. This file was imported into Cytoscape software, where a PPI network was constructed and visualized. Topological parameters, including node degree, were analyzed using Cytoscape's network analysis plug‐in, with higher‐degree nodes identified as critical targets in IRN's action mechanism on osteoporosis.

The DAVID database (https://david.ncifcrf.gov/tools.jsp) was used to perform Gene Ontology (GO) and Kyoto Encyclopedia of Genes and Genomes (KEGG) enrichment analyses on the intersection target genes. The GO analysis covered biological processes (BP), cellular components (CC), and molecular functions (MF), while KEGG pathway enrichment was conducted with species set to “ *Homo sapiens* ” and significance set at *p* < 0.05. For visualization, a bubble chart and histogram were generated using the bioinformatics platform (http://www.bioinformatics.com.cn/).

### 2.2 Molecular Docking

To investigate the binding affinity and interaction of IRN with the key target proteins associated with osteoporosis, molecular docking was performed using Schrödinger's Maestro software suite. Initially, the 3D structures of core target proteins were retrieved from the Protein Data Bank (PDB, https://www.rcsb.org/) in PDB format. Protein preparation was conducted in Maestro, which included removal of water molecules, addition of hydrogen atoms, and optimization of bond orders to ensure accuracy in docking. For IRN, the molecular structure was obtained in 2D format from the PubChem database (https://pubchem.ncbi.nlm.nih.gov/), converted to 3D format, and prepared using the LigPrep module in Maestro. Energy minimization was performed to optimize the ligand geometry, ensuring the most stable conformations for docking. The receptor grid was generated around the active site of each protein to define the binding region. Molecular docking was conducted using the Glide module, where both standard precision (SP) and extra precision (XP) modes were applied to ensure reliability of the binding poses and affinities. Docking scores, indicative of binding energy and stability, were calculated for each target‐ligand interaction, with lower scores suggesting stronger binding affinities. The resulting binding interactions were visualized in Maestro, highlighting hydrogen bonding, hydrophobic interactions, and other key contacts between IRN and the target residues within the binding pocket.

### 2.3 Animal and Materials

#### 2.3.1 Animal Models

The animal study protocol was approved by the Ethics Committee of The First Affiliated Hospital of Guangdong Pharmaceutical University (Approval No. 2024005), in compliance with the Guide for the Care and Use of Laboratory Animals. A total of 28 specific pathogen‐free (SPF) grade, 8‐week‐old female C57BL/6J mice were obtained from Guangdong Medical Laboratory Animal Center and randomly divided into four groups (*n* = 7 per group): Sham, OVX, IRN‐L (10 mg/kg), and IRN‐H (20 mg/kg). The doses of IRN were determined based on previous studies using cell‐based experiments, ensuring the effective and safe range for the animal model (Li et al. 2022; Wu, Bian, et al. 2023). All mice were housed under identical conditions in an SPF‐grade facility, with free access to water and a standard diet. Following 1 week of acclimatization, mice in OVX, IRN‐L, and IRN‐H underwent bilateral ovariectomy under general anesthesia via intraperitoneal injection of 2% sodium pentobarbital (30 mg/kg). Sham‐operated mice underwent a similar procedure, but only the adipose tissue surrounding the ovaries was removed. After a 1‐week recovery period, IRN‐L and IRN‐H received intragastric administration of IRN suspended in 0.5% carboxymethylcellulose sodium (CMC‐Na) solution at doses of 10 and 20 mg/kg, respectively, once daily for 8 weeks. Mice in OVX and Sham were administered an equivalent volume of physiological saline containing 0.5% CMC‐Na. The body weight of the mice was monitored weekly, and the dosage was adjusted accordingly. At the end of the 8‐week treatment, left femurs, colon tissue, serum, and fecal samples were collected for further analysis.

#### 2.3.2 Reagents

IRN (purity > 98%) was purchased from Chengdu Must Biotechnology Co. Ltd., Chengdu, China. The enzyme‐linked immunosorbent assay (ELISA) kits for detecting tumor necrosis factor‐alpha (TNF‐α), interleukin‐1 beta (IL‐1β), interleukin‐6 (IL‐6), interleukin‐10 (IL‐10), tartrate‐resistant acid phosphatase 5b (TRACP5b), beta‐C‐terminal telopeptide of type I collagen (β‐CTX), procollagen type I N‐terminal propeptide (P1NP), bone alkaline phosphatase (BALP), nitric oxide (NO), inducible nitric oxide synthase (iNOS) and reactive oxygen species (ROS) were obtained from Jiangsu Meimian Industrial Co. Ltd., Jiangsu, China. Primary antibodies against Zonula Occludens‐1 (ZO‐1), Occludin, and Claudin‐1, as well as the horseradish peroxidase (HRP)‐conjugated secondary antibodies, were sourced from Beyotime Biotechnology, China.

### 2.4 Micro‐CT Scanning

The left femurs of all mice were carefully dissected, cleared of any adhering soft tissues, and immediately fixed in 4% paraformaldehyde for 48 h. Micro‐CT analysis was performed using the SCANCO μCT 100 scanner (Hangzhou Yue Bo Biological Technology Co. Ltd., Hangzhou, China). Scanning parameters were set as follows: a tube voltage of 70 kV, an electric current of 200 μA, an isotropic voxel size of 10 μm, and an exposure time of 300 ms. For the analysis, the region located 0.5 mm to 0.8 mm from the distal femur towards the growth plate was defined as the Region of Interest (ROI). The 3D reconstruction and analysis of bone microarchitecture were performed using Evaluation V6.5‐3 software (SCANCO Medical AG, Switzerland). Key bone parameters were quantified, including bone mineral density (BMD), bone volume fraction (BV/TV), trabecular number (Tb.N), trabecular thickness (Tb.Th), and trabecular separation (Tb.Sp).

### 2.5 Analyses of Biochemical Markers, Inflammatory Mediators, and Bone Metabolism Indicators

ELISA kit was used to quantitatively determine the serum levels according to the manufacturer's protocol. Briefly, blood samples were collected from the retro‐orbital sinus, allowed to clot at room temperature, and centrifuged at 3000 rpm for 15 min to separate the serum. The serum was aliquoted and stored at −80°C until analysis.

Each biomarker was assayed using specific ELISA kits: TNF‐α, IL‐1β, IL‐6, and IL‐10 were measured to evaluate systemic inflammatory responses; TRACP5b and β‐CTX as indicators of bone resorption activity; P1NP and BALP as markers of bone formation; NO and iNOS to monitor oxidative stress levels; ROS as a marker of oxidative damage. The absorbance for each assay was measured using a microplate reader at the recommended wavelengths, and the concentrations were calculated based on standard curves generated from known concentrations of the respective analytes. All samples were assayed in duplicate to ensure accuracy and reproducibility.

### 2.6 Mice Colons Histomorphometric and Immunohistological Analysis

Fresh colon tissues were harvested, fixed in 10% formalin for 24 h, processed through a graded ethanol series, embedded in paraffin, and sectioned into 3 μm thick slices. Hematoxylin and eosin (H&E) staining was performed to evaluate the pathological changes in the colon tissue. Colon sections were dewaxed, dehydrated, antigen‐repaired, and endogenous peroxidase activity was blocked with 3% hydrogen peroxide, followed by washing in phosphate‐buffered saline (PBS). Sections were incubated at room temperature for 30 min with primary antibodies against ZO‐1 (1:2400), Occludin (1:2400), and Claudin‐1 (1:200). After washing with PBS, the sections were incubated with HRP‐conjugated secondary antibodies for 20 min. DAB chromogen was applied for 5 min, followed by counterstaining with hematoxylin for 30 s. The stained sections were rinsed under running water for 5 min and examined under a light microscope. The expression levels of ZO‐1, Occludin, and Claudin‐1 were quantified by measuring the mean optical density (MOD) using Image J software.

### 2.7 16S rRNA Gene Sequencing

Fecal samples were collected in sterile 1.5 mL Eppendorf tubes and immediately stored at −80°C until further processing. Genomic DNA was extracted using the E.Z.N.A. Soil DNA Kit (Omega Bio‐tek, Norcross, GA, USA), following the manufacturer's protocol. The quality of the extracted DNA was assessed by 1% agarose gel electrophoresis, and the concentration and purity of the DNA were measured using a NanoDrop 2000 spectrophotometer (Thermo Scientific, USA). The V3‐V4 hypervariable regions of the 16S rRNA gene were amplified by polymerase chain reaction (PCR) using primers with barcode sequences. The forward primer was 338F (5′‐ACTCCTACGGGAGGCAGCAG‐3′) and the reverse primer was 806R (5′‐GGACTACHVGGGTWTCTAAT‐3′). PCR amplification was carried out with TransStart FastPfu DNA Polymerase (TransGen Biotech, Beijing, China) to ensure high‐fidelity amplification, and each sample was amplified in triplicate. The resulting PCR products were confirmed by 2% agarose gel electrophoresis, and the AxyPrep DNA Gel Extraction Kit (Axygen, USA) was used for purification. Quantification of the purified PCR products was performed using the QuantiFluor‐ST Fluorometer (Promega, USA). The amplicon library was prepared using the TruSeq DNA Sample Preparation Kit (Illumina, USA) following the manufacturer's instructions, and sequencing was conducted on the Illumina MiSeq PE300 platform (Shanghai Majorbio Bio‐Pharm Technology Co. Ltd., Shanghai, China). Paired‐end reads were generated, and further analyses were carried out to profile the bacterial composition of each sample.

### 2.8 SCFAs Quantification Analysis

SCFAs were extracted and analyzed using gas chromatography–mass spectrometry (GC–MS). A 20 mg fecal sample was accurately weighed into a 2 mL grinding tube, followed by the addition of 800 μL of 0.5% phosphoric acid solution containing 10 μg/mL of the internal standard 2‐ethylbutyric acid. The samples were subjected to cold grinding for 3 min at 50 Hz, followed by ultrasonic extraction for 10 min. The mixture was then centrifuged at 13,000 g at 4°C for 15 min. The resulting supernatant (200 μL) was transferred to a 1.5 mL centrifuge tube and mixed with 200 μL of n‐butanol solvent for extraction. After vortexing for 10 s and ultrasonication at 4°C for another 10 min, the sample was centrifuged again at 13,000 g for 5 min. The final supernatant was transferred to a gas chromatography vial for analysis. GC–MS analysis was performed using an Agilent 8890B‐5977B GC/MS system (Agilent Technologies, USA) equipped with a HP‐FFAP capillary column (30 m × 0.25 mm × 0.25 μm). The carrier gas was helium (99.999% purity) with a flow rate of 1 mL/min. The injection volume was 1 μL in split mode (10:1). The temperature of the column oven was initially set to 80°C, then increased to 120°C at 20°C/min, followed by a rise to 160°C at 5°C/min, and finally held at 220°C for 3 min. The mass spectrometer was operated in electron impact ionization (EI) mode at 70 eV. The ion source and quadrupole temperatures were set to 230°C and 150°C, respectively. Data acquisition was performed in selective ion monitoring (SIM) mode. SCFAs quantification was conducted using the Masshunter software (Agilent Technologies) and SCFAs concentrations were calculated based on calibration curves derived from known standards.

### 2.9 Statistical Analysis

Data are presented as mean ± standard deviation (SD). Normality of the data was assessed using the Shapiro–Wilk test. For datasets following a normal distribution, one‐way analysis of variance (ANOVA) was employed, followed by Tukey's post hoc test for multiple comparisons. Non‐normally distributed data were analyzed using the Mann–Whitney *U* test. Statistical analyses were performed using *SPSS* software (version 27.0, IBM Corp., Armonk, NY, USA). Results from 16S rRNA gene sequencing were analyzed through the Majorbio Cloud Platform (https://cloud.majorbio.com), with significance set at *p* < 0.05.

## 3 Results

### 3.1 Network Pharmacology and PPI Network Analysis

**FIGURE 1:**
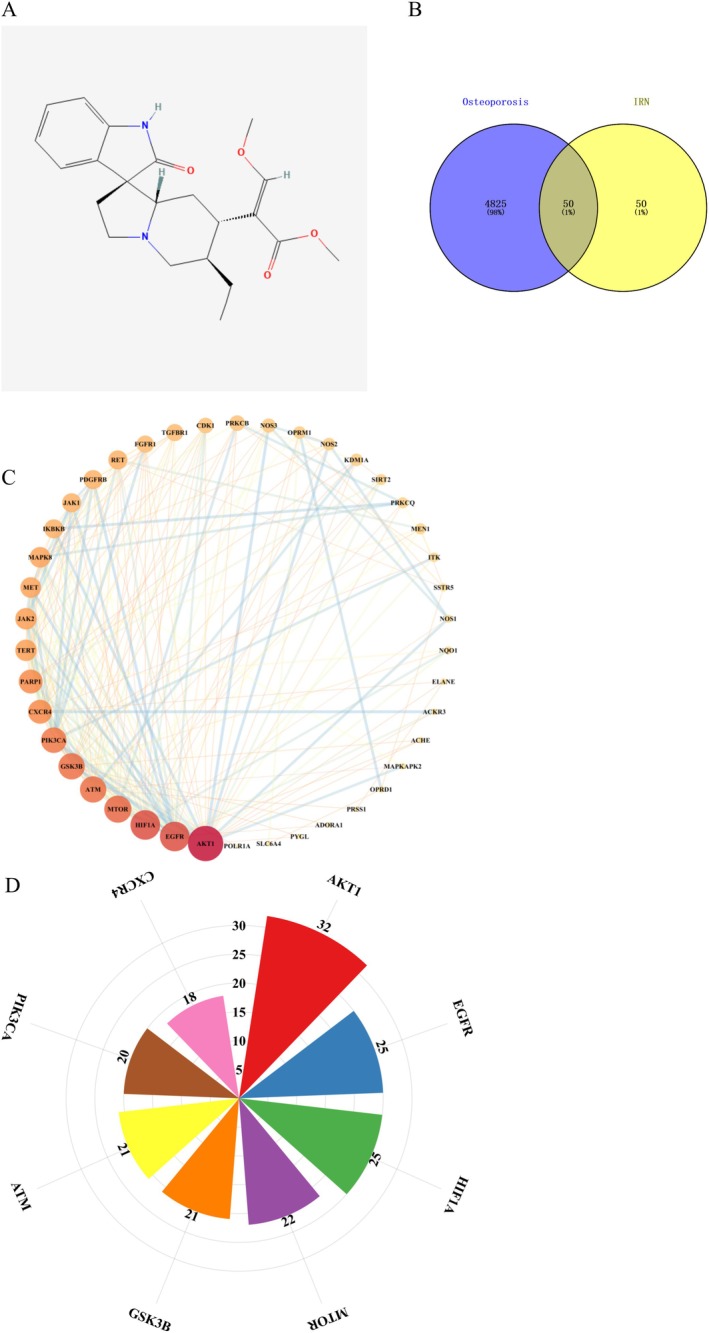
Network pharmacology and PPI network analysis. (A) The chemical structure of IRN was provided by the PubChem database (CID: 3037048). (B) Venn diagram of osteoporosis‐related targets and IRN‐related targets. (C) PPI network of IRN‐OP common target genes. (D) Ranking the top 8 targets in the PPI network in degree.

Network pharmacology analysis identified 100 potential targets of IRN (Figure 1A) and 4875 osteoporosis (OP)‐related targets, where 50 overlapping genes are potential targets for the treatment of OP using IRN (Figure 1B). To delve deeper into the relationships among these selective targets, a protein–protein interaction (PPI) network was constructed using the Cytoscape platform (Figure 1C). Determined by node degree, the top 8 hub genes include AKT1, EGFR, HIF1A, MTOR, GSK3B, ATM, PIK3CA, and CXCR4, which may play crucial roles in the therapeutic effects of IRN on OP (Figure 1D).

### 3.2 GO and KEGG Pathway Enrichment Analysis

**FIGURE 2:**
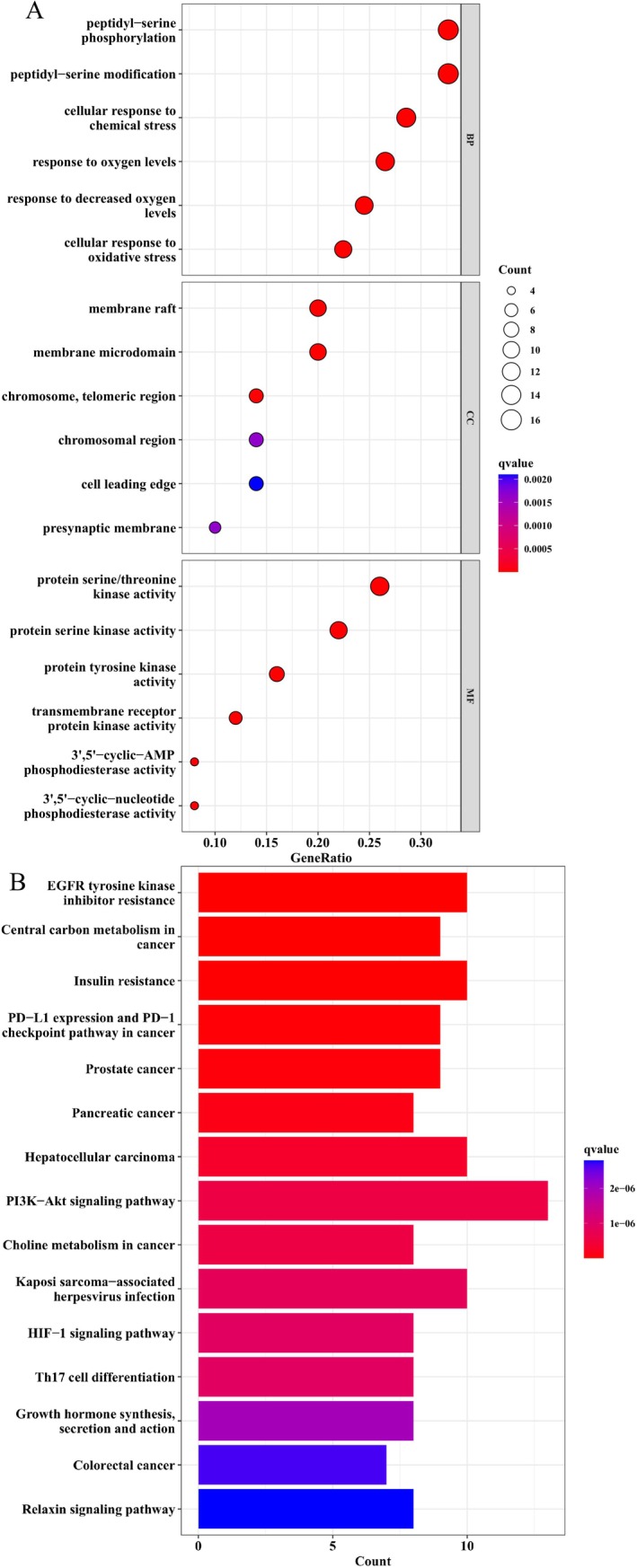
GO and KEGG pathway enrichment analysis. (A) Analysis of GO enrichment BP, CC, and MF items from the DAVID database. (B) Analysis of the KEGG pathways about IRN anti‐OP from the DAVID database.

To further elucidate the mechanisms of IRN on treating OP, GO and KEGG enrichment analyses were conducted to interpret gene sets derived from network pharmacology and PPI analysis. The GO enrichment analysis revealed BP, CC, and MF highly associated with IRN targets (Figure 2A). Key BP like the “cellular response to oxidative stress” and “response to decreased oxygen levels” are likely affected by IRN. IRN's impact also influences CC such as the “chromosomal region” and “cell leading edge.” Furthermore, IRN's target genes are implied to be involved in MF, e.g., “protein serine/threonine kinase activity” and “transmembrane receptor protein kinase activity.” In terms of KEGG pathway analysis, IRN's target genes are enriched primarily in pathways related to bone health and disease regulation (Figure 2B), where the PI3K‐Akt signaling pathway that controls cell survival and proliferation is most likely activated to exert therapeutic effect upon IRN treatment. Other pathways, such as the HIF‐1 signaling pathway and Th17 cell differentiation, may represent synergistic activities contributing to IRN's curative effect. These results indicate the multi‐target properties of IRN and its potential impact on pathways associated with oxidative stress, inflammation, and osteoporosis.

### 3.3 Molecular Docking

**FIGURE 3:**
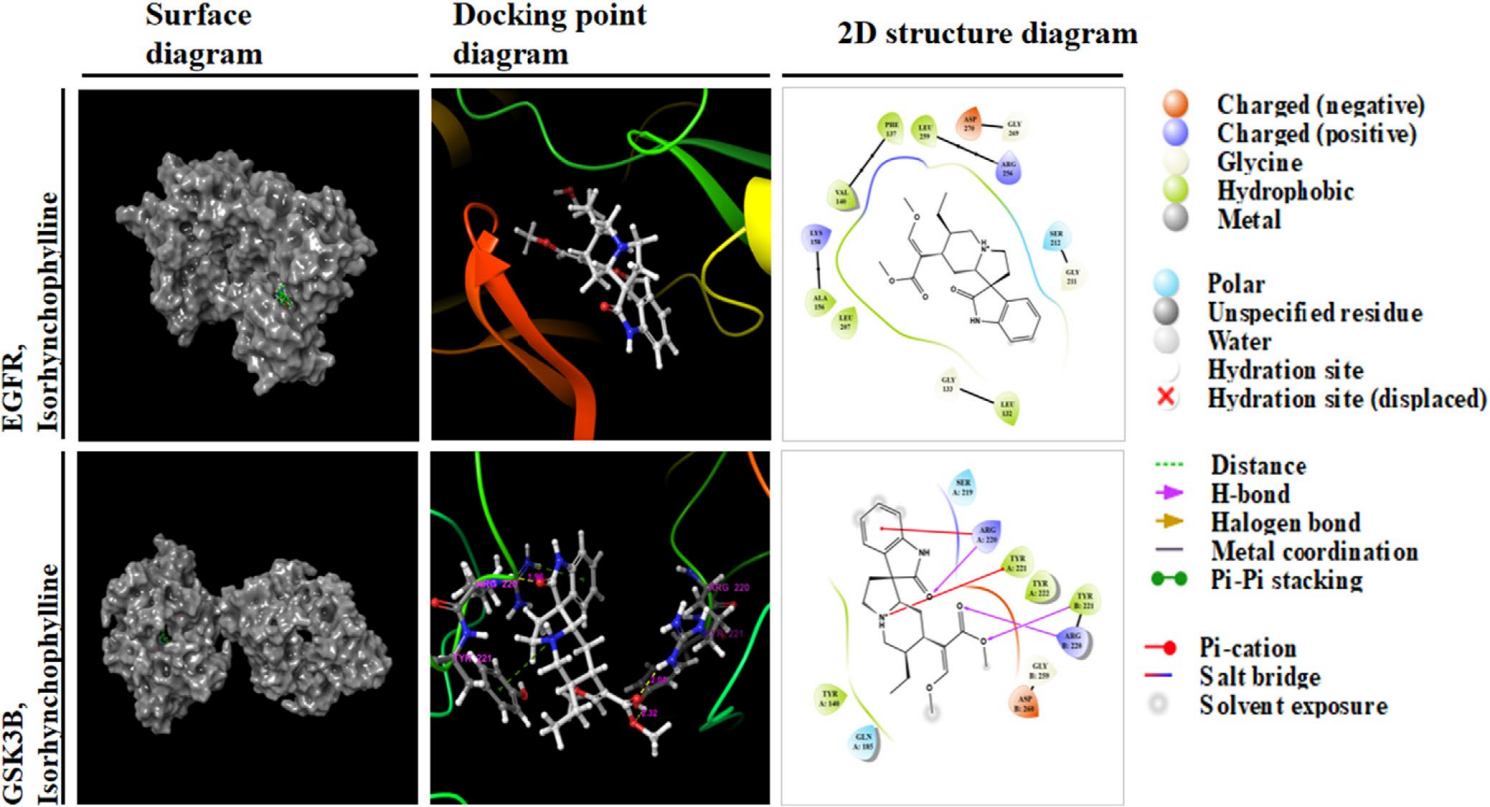
Molecular docking. Molecular docking visualization of IRN with the top two core target proteins, EGFR and GSK3B.

To understand the molecular interactions between IRN and its top‐ranked in silico interactive partners, EGFR and GSK3B were docked to IRN using Schrödinger's Maestro software. The docking scores were −5.433 kcal/mol for EGFR and −5.179 kcal/mol for GSK3B, indicating strong binding affinities (Table 1). Visualization of the docking results revealed that IRN interacts with key residues of EGFR, including LEU 132 and ARG 256 through hydrogen bonding and hydrophobic interactions. Similarly, IRN forms stable interactions with GSK3B, engaging residues such as ARG 220 and TYR 221 via hydrogen bonding and pi‐pi stacking (Figure 3). These specific binding sites and interaction types underline the potential molecular mechanisms of IRN's activity against EGFR and GSK3B.

**TABLE 1:** Docking results of IRN with EGFR and GSK3B.

Target	PDB ID	Affinity (kcal/mol)
EGFR	3EQP	−5.433
GSK3B	4J1R	−5.179

### 3.4 IRN Alleviated OVX‐Induced Bone Loss

**FIGURE 4:**
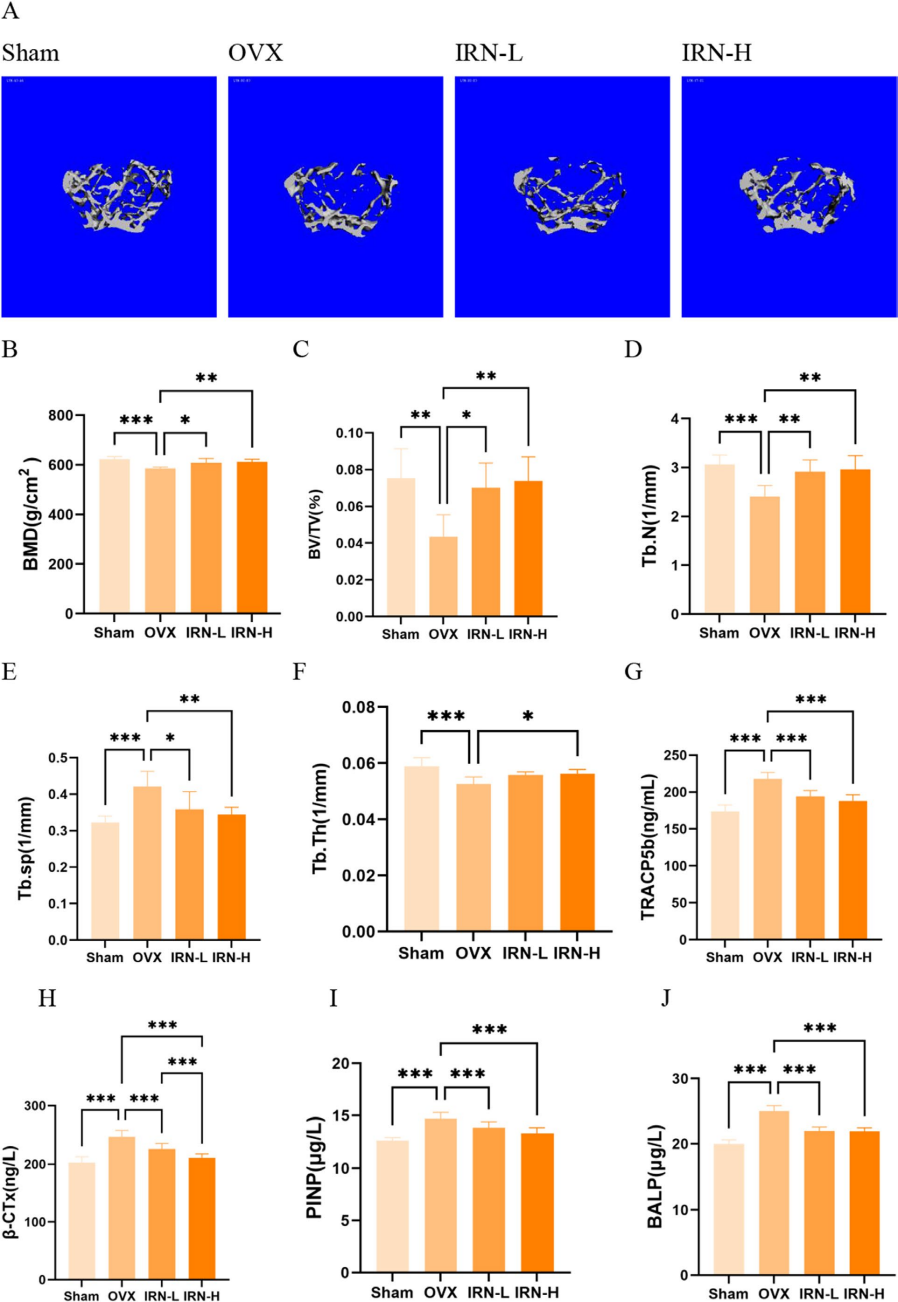
IRN treatment attenuated OVX‐induced bone loss. (A) Representative images of mouse femur micro‐CT (scale bar = 100 μm). (B–F) Micro‐CT quantitative parameters for bone microstructure including BMD, BV/TV, Tb.N, Tb.Sp, and Tb.Th. (G–J) The levels of TRACP5b, β‐CTX, P1NP, and BALP in serum. Data are presented as the mean ± SD (*n* = 7); *p < 0.05, **p < 0.01, and ***p < 0.001.

To examine the in vivo protective effect of IRN against excessive bone loss, ovariectomized (OVX) mice were treated for 8 weeks with either 10 mg/kg IRN (IRN‐L), 20 mg/kg IRN (IRN‐H), or an equal volume of saline. The results were compared with those of the sham group. Micro‐CT images showed a markedly reduced bone volume in trabeculae in OVX mice in comparison with sham mice, characterized by sparse and discontinuous trabeculae (Figure 4A). Treatment with IRN for 8 weeks improved the compromised trabecular structure induced by ovariectomy, where IRN‐L and IRN‐H groups showed improved trabecular connectivity and density in a dose‐dependent manner. Quantitatively, BMD, BV/TV, and Tb.N were evidently decreased in OVX mice, which was significantly reversed by IRN intervention (Figure 4B–D). On the other hand, Tb.Sp and Tb.Th were increased in OVX mice, indicating poor trabecular architecture and bone health (Figure 4E,F). IRN treatment alleviated the osteoporotic symptoms and enhanced the quality of the trabecular bone (Figure 4E,F). To further confirm the efficacy of IRN, serum levels of bone metabolism markers were also measured. Serum levels of TRACP5b, β‐CTX, P1NP, and BALP in the OVX were also markedly elevated compared with the sham, while decreased after treatment by IRN, indicating its potential to restore skeletal homeostasis by suppressing excessive bone resorption and formation (Figure 4G–J). We provided the firsthand evidence that shows the protective effect of IRN against OVX‐induced bone loss, representative of a novel anti‐osteoporosis option.

### 3.5 IRN Modulates Inflammation and Oxidative Stress in OVX Mice

**FIGURE 5:**
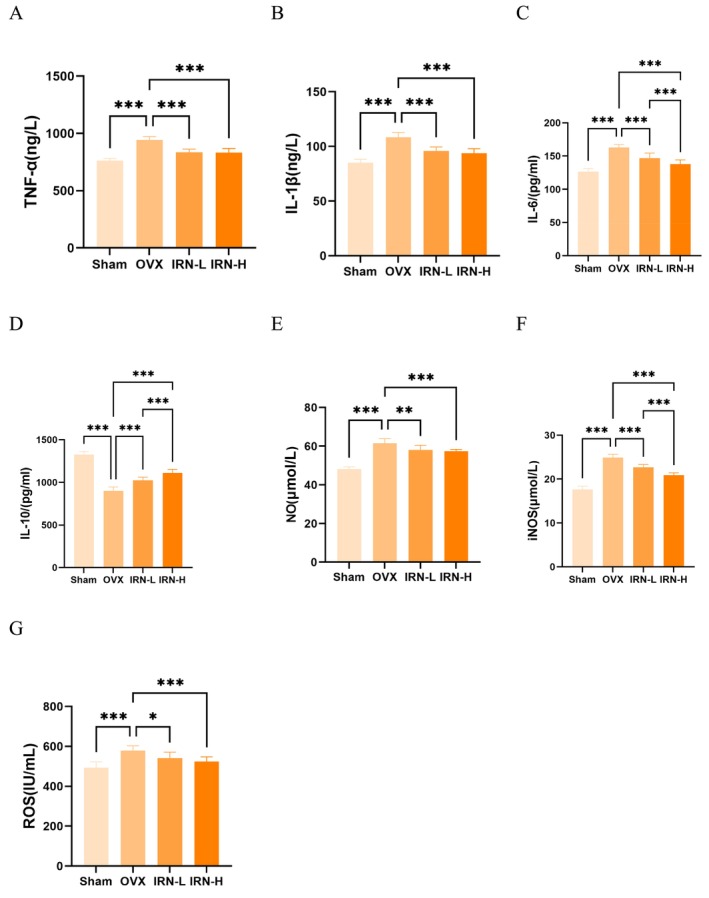
IRN modulates inflammation, oxidative stress, and bone remodeling in OVX mice. (A–C) The serum levels of inflammatory cytokines TNF‐α, IL‐1β, and IL‐6 in serum. (D) The levels of anti‐inflammatory cytokine IL‐10. (E–G) The serum levels of NO, iNOS, and ROS in serum. Data are presented as the mean ± SD (*n* = 7). *p < 0.05, **p < 0.01, and ***p < 0.001.

Inflammation is closely related to skeletal homeostasis. Inflammatory cytokine levels were also seen markedly altered in OVX mice and can be reversed by IRN treatment. Serum levels of TNF‐α, IL‐1β and IL‐6 were significantly elevated in OVX compared to the Sham, in which the increase of these inflammatory cytokines was alleviated by IRN‐L and IRN‐H treatments (Figure 5A–C). We further examined the anti‐inflammatory cytokine (Figure 5D), IL‐10, which was significantly reduced by ovariectomy and rescued by IRN‐L and IRN‐H treatments in a dose‐dependent manner.

Oxidative stress plays a significant role in the pathophysiology of bone loss associated with ovariectomy in mice. Oxidative stress markers NO, iNOS, and ROS (Figure 5E–G) were consistently elevated in OVX mice. Treatments with IRN‐L and IRN‐H achieved a significant reduction in NO levels. Accordingly, IRN treatment dose‐dependently eliminated the excessive levels of iNOS and ROS, indicative of an effective protection mediated by IRN against inflammation and oxidative stress, demonstrating the therapeutic potential of IRN.

### 3.6 IRN Repairs Intestinal Barrier Function and Modulates Gut Microbiota to Enhance SCFAs Production

**FIGURE 6:**
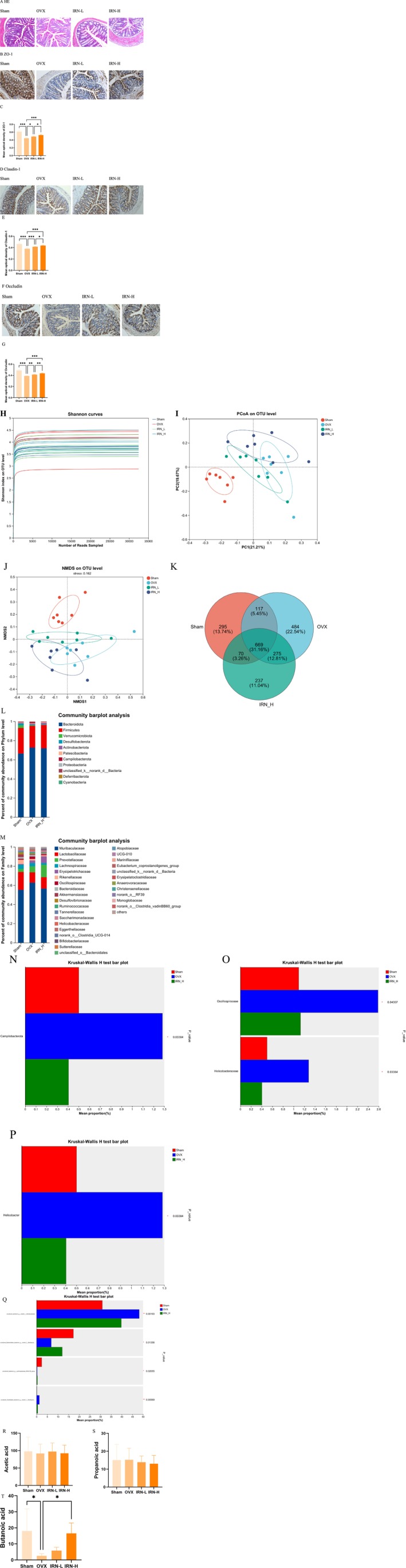
Effects of IRN on colonic inflammation, intestinal integrity, barrier‐related protein expression, gut microbiota composition, and SCFAs production in OVX mice. (A) HE staining of colon tissue (magnification, 10×). (B) ZO‐1, (D) Claudin‐1, and (F) Occludin stain the colonic tissue sections (magnification, 10×). Expression of tight junction proteins ZO‐1 (C), Claudin 1 (E), and Occludin (G). (H) Shannon curves. (I) PCoA on OTU level. (J) NMDS on OTU level. (K) The OTU data was represented by Venn diagram. (L) Analysis of microbial communities at the phylum level. (M) Analysis of microbial communities at the family level. (N) Phylum (O) family (P) genus (Q) *speciese*. In the column diagram, the red bar represents Sham, the blue bar represents OVX, and the green bar represents IRN‐H. IRN increased SCFAs production present in the feces. (R) Acetic acid, (S) Propanoic acid, (T) Butanoic acid. Data are presented as the mean ± SD (*n* = 7). *p < 0.05, **p < 0.01, and ***p < 0.001.

Ovariectomy leads to estrogen deficiency and elevated inflammatory responses, which are associated with increased intestinal permeability due to the disruption of the intestinal barrier function by gut microbiota. HE staining showed significant changes in intestinal morphology in OVX mice, with a sparse intestinal cavity, enlarged epithelial space, and obvious infiltration of inflammatory cells, indicating deteriorated intestinal barrier structure (Figure 6A). Furthermore, immunohistochemical analysis revealed that the expression levels of tight junction proteins like ZO‐1, Claudin‐1, and Occludin were significantly decreased in OVX mice, suggesting increased intestinal permeability and compromised barrier function (Figure 6B–G). IRN treatment effectively repaired those OVX‐induced intestinal barrier damage (Figure 6A). Both IRN‐L and IRN‐H treatment groups showed improved epithelial continuity, narrow intestinal lumen space, reduced inflammatory cell infiltration, and enhancement of tight junction protein expression, where a strong protective effect was noted with IRN‐H treatment (Figure 6B–G) (*p* < 0.05, *p* < 0.01, *p* < 0.001).

As mentioned, gut microbiota and their metabolites are highly associated with skeletal homeostasis. To evaluate the impact of IRN on gut microbiota composition and diversity in OVX mice, 16S rRNA sequencing was performed. Shannon diversity curves refer to sufficient sequencing depth (Figure 6H). Principal Coordinate Analysis (PCoA) along with Non‐metric Multidimensional Scaling (NMDS) based on Bray‐Curtis distances revealed distinct microbial community structures between different groups, highlighting IRN's influence on microbial diversity (Figure 6I,J). IRN‐L did not significantly restore the OVX‐induced microbiota imbalance (data are not shown), so subsequent analysis focused on the Sham, OVX, and IRN‐H. Venn diagram analysis identified 669 operational taxonomic units (OTUs) across different groups, with 295 unique to the Sham group, 484 unique to the OVX group, and 237 unique to the IRN‐H (Figure 6K). We analyzed microbial communities at the phylum and family levels (Figure 6L,M). OVX increased the relative abundance of *Bacteroidota* and decreased *Firmicutes* at the phylum level, while at the family level, it elevated the relative abundance of *Muribaculaceae* and reduced *Lactobacillaceae*. IRN‐H reversed these changes. To further investigate how IRN regulates the imbalance in the gut microbiota of OVX mice, we analyzed microbial differences between the Sham, OVX, and IRN‐H at the phylum, family, genus, and species levels. At the phylum level, OVX increased the abundance of *Campylobacterota*, while IRN‐H treatment reduced its abundance close to Sham's level (Figure 6N). A similar trend was also observed in the analysis at the family level, where the increases of *Helicobacteraceae* and *Oscillospiraceae* in OVX were evidently restored by IRN‐H treatment (Figure 6O). The analysis at the genus level showed that IRN‐H treatment reduced the level of *Helicobacter*, which was otherwise elevated following ovariectomy (Figure 6P). At the species level, IRN‐H treatment reduced the level of *uncultured_bacterium_g__norank_f__Muribaculaceae* and *uncultured_Clostridiales_bacterium_g__norank_f__Oscillospiraceae*; whereas, it improved the level of *uncultured_Bacteroidales_bacterium_g__norank_f__Muribaculaceae* and *uncultured_bacterium_g__Lachnospiraceae_NK4A136_group* (Figure 6Q).

SCFAs are metabolites produced by gut microbiota, which are largely affected by intestinal barrier function and the composition of gut microbiota. GC–MS analysis of fecal SCFAs showed no significant differences in the concentrations of acetic acid and propionic acid between the OVX and Sham, nor following IRN treatment (Figure 6R,S). However, the concentration of fecal butanoic acid was significantly lower in OVX compared to the Sham, in which the tendency was reversed by IRN treatment (Figure 6T). SCFAs, especially butyric acid, are essential for bone metabolism because they promote calcium absorption, regulate the activity of osteoblasts and osteoclasts, and suppress inflammation throughout the body, thereby preventing bone loss.

Collectively, the IRN treatment demonstrated a significant impact on SCFAs, gut microbiota composition, and diversity in OVX mice, including the restoration of decreased SCFAs, the reduction of fecal butanoic acid levels, and an adjustment in the microbial community structure. Notably, IRN treatment effectively reversed the OVX‐induced increases in the abundance of *Campylobacterota*, *Helicobacteraceae*, and *Oscillospiraceae*, demonstrating its potential to regulate gut microbiota imbalance. These findings suggest that IRN plays a protective role against OVX‐induced bone loss by repairing the gut barrier and regulating gut microbiota composition, ultimately boosting SCFAs production.

## 4 Discussion

OP is a systemic skeletal disorder characterized by reduced BMD and microarchitectural deterioration of bone tissue, leading to diminished bone strength and increased bone fragility (Johnston and Dagar 2020). PMOP is the most prevalent form of OP, majorly due to estrogen decline following menopause. This hormonal imbalance triggers an excessive inflammatory response, manifested by elevated pro‐inflammatory cytokines, such as IL‐1β and IL‐6, which fuel bone resorption and hamper bone formation (Cheng et al. 2022; Xu et al. 2024; Hsu et al. 2024). At the same time, estrogen deficiency can induce the imbalance of gut microbiota and the impairment of intestinal barrier function, thereby increasing intestinal permeability, accelerating the inflammatory response, and ultimately aggravating osteoporosis (Zhang et al. 2022). SCFAs, including acetic acid, propanoic acid, and butanoic acid, are metabolites of gut microbiota fermentation and decomposition of some carbohydrates and amino acids (Fusco et al. 2023). These metabolites maintain the integrity of the intestinal barrier by promoting the differentiation of intestinal epithelial cells and the expression of tight junction proteins such as ZO‐1 and Occludin, thereby reducing bacterial translocation and inflammatory responses, which are key factors in the development of osteoporosis (Feng et al. 2024). This study demonstrated the therapeutic potential of IRN on PMOP and has systematically investigated its protective effect in OVX mice through examining the changes of inflammatory responses, antioxidants, gut microbiota, and SCFAs.

Network pharmacological analysis revealed that PI3K‐Akt is a critical signaling pathway potentially mediated by IRN treatment. The PI3K‐Akt signaling pathway is a key signal transduction pathway involved in bone metabolism and bone remodeling, which is closely related to the proliferation and differentiation of osteoblasts and osteoclasts (Zuo et al. 2022). Specifically, the PI3K‐Akt signaling pathway can activate mTOR, promote protein synthesis, and reduce osteoblast apoptosis caused by inflammatory response and oxidative stress. At the molecular level, increasing the phosphorylation levels of PI3K and AKT proteins can promote the proliferation and differentiation of osteoblasts (Liu et al. 2025). Previous studies have demonstrated the efficacy of traditional Chinese medicine monomers on the PI3K‐Akt signaling pathway in various BP. Luteolin has been found to alleviate osteoblast pyroptosis through the activation of the PI3K‐AKT signaling pathway (Chai et al. 2024). Similarly, naringin has been found to stimulate the PI3K‐Akt pathway, thereby promoting osteogenic differentiation (Wang, Liang, et al. 2024). Molecular docking analysis suggested that, in addition to PI3K‐Akt, IRN potentially targets EGFR and GSK3B, which, in turn, can reduce inflammatory response. EGFR is a key regulator in bone remodeling. EGFR signaling activation can up‐regulate the expression of early growth response protein 2 and promote the proliferation of osteoprogenitor cells (Mu et al. 2025); however, overactivation of EGFR signaling promotes the secretion of inflammatory cytokines such as IL‐1β and IL‐6, leading to increased osteoclast activity and bone resorption (Linder et al. 2018). GSK3B signaling activation can promote the proliferation and differentiation of osteoblasts (Rong et al. 2022), in which overactivation of it can also upregulate pro‐inflammatory cytokines, thereby fueling osteoclast activity and exacerbating bone loss (Xu et al. 2019). IRN may play a positive role in the regulation of bone metabolism by activating the PI3K‐Akt signaling pathway and inhibiting the excessive activation of EGFR and GSK3B to reduce the activation of osteoclasts.

In vivo, IRN significantly decreased the serum levels of pro‐inflammatory cytokines such as TNF‐α, IL‐1β, and IL‐6. Concurrently, it also increased the levels of the anti‐inflammatory cytokine IL‐10, as evidenced by serum biomarker analysis. TNF‐α influences the onset and progression of osteoporosis by inhibiting osteoblast differentiation through immunoinflammation (Zhang et al. 2020). IL‐1β, a pro‐inflammatory factor, promotes the production of RANKL, which fuels osteoclast differentiation and bone resorption (Nakamura and Jimi 2006). IL‐6, produced by bone marrow stromal cells, accelerates bone resorption by driving osteoclast differentiation and function via RANKL expression (Hashizume et al. 2008). Meanwhile, IL‐10, an immunomodulator with strong anti‐inflammatory properties, can directly inhibit osteoclast differentiation and enhance osteoblast proliferation (Ouyang et al. 2011). These together evidence the beneficial potential of IRN on bone health.

Bone metabolism is a dynamic process that maintains homeostasis between bone resorption and formation (Ouyang et al. 2011). TRACP5b and β‐CTX are markers of bone resorption. TRACP5b is an enzyme secreted by osteoclasts, indicating the differentiation and activity of osteoclasts (Chao et al. 2010). β‐CTX is a product of P1NP, which reflects the degree of collagen degradation during bone resorption (Wei et al. 2021). On the other hand, P1NP and BALP are bone formation markers, which can be used to monitor osteoblast activity and matrix mineralization (Konukoğlu 2019). In the current study, the increases in serum TRACP5b and β‐CTX induced by ovariectomy were alleviated by IRN treatment. This is accompanied by increased bone mass, featured by improved BMD, BV/TV, Tb.N, Tb.Th, and Tb.Sp. Interestingly, P1NP and BALP levels were also elevated in OVX compared to the Sham, likely representing a compensatory effect, although further investigation is required to confirm this phenomenon (Eastell et al. 2021). IRN treatment notably decreased the levels of P1NP and BALP in OVX mice, indicating the improvement of bone health and re‐achievement of skeletal homeostasis.

Oxidative stress disrupts bone metabolism and is an important contributor to OP (Tan et al. 2018). ROS hampers the turnover between bone resorption and bone formation (Marques‐Carvalho et al. 2023). NO is synthesized by iNOS, and excessive NO production exacerbates osteoclast activity and bone resorption (Herrera et al. 2011). We demonstrated that IRN profoundly alleviated oxidative stress with reduced serum levels of NO, iNOS, and ROS and improved bone microarchitecture in OVX mice. This reduction aligns with findings on other Chinese medicine monomers, including resveratrol, curcumin, and quercetin, which have also been shown to scavenge ROS and downregulate iNOS expression, mitigating bone loss and remitting osteoporotic phenotypes (Zhou et al. 2019; Li, Chen, Mao, et al. 2020; Wang et al. 2021).

The gut microbiota, a newly emerging field closely linked to bone metabolism, influences host metabolism through SCFAs (Li et al. 2019). 16S rRNA sequencing analysis revealed that IRN treatment modulated gut microbiota composition in OVX mice; in particular, it reduced pathogenic taxa and increased the populations of beneficial gut bacteria. *Campylobacterota* is a potential pathogen; studies have shown that the occurrence and development of PMOP are related to the increase of *Campylobacterota* abundance (Ma et al. 2024). *Helicobacter* reduction may suppress the secretion of inflammatory cytokines and mitigate chronic inflammation, a known contributor to OP (Brackman et al. 2024). *norank_f__Muribaculaceae* belongs to the family *Muribaculaceae* within the phylum *Bacteroidetes. Muribaculaceae* cause chronic intestinal inflammation and intestinal mucosal barrier disruption (Zhou et al. 2024). *Lachnospiraceae_NK4A136* is a beneficial bacterium that can produce butyric acid; studies have shown that it can alleviate inflammatory bowel disease (IBD) through its anti‐inflammatory capacity (Zhou et al. 2022). Although direct evidence linking *Helicobacter*, *Muribaculaceae*, and *Lachnospiraceae_NK4A136* to bone metabolism is limited, IRN may reduce inflammation and repair the intestinal barrier by reducing the abundance of them.

On the other hand, butanoic acid and propanoic acid can induce metabolic reprogramming of osteoclasts, leading to enhanced glycolysis and reduced oxidative phosphorylation, thereby down‐regulating key genes in osteoclasts, providing a basis for a direct mechanistic link between gut microbiota and bone (Lucas et al. 2018; Deng et al. 2024). Furthermore, butanoic acid can enhance the tight junction of intestinal epithelial cells and calcium absorption, thereby reducing the invasion and colonization of the intestinal tract by pathogenic bacteria and the remission of OVX‐induced excessive bone loss (Ferrer‐Picón et al. 2020). In OVX mice, IRN treatment notably increased butanoic acid abundance, although it did not significantly affect the levels of acetic acid and propanoic acid. This all suggests that IRN may alter gut microbiota composition to promote the secretion of butanoic acid, which reduces the production of pathogenic bacteria and increases the production of beneficial bacteria, thereby improving bone metabolism. Additionally, immunohistochemical analysis revealed upregulation of key tight junction proteins, such as ZO‐1, Claudin‐1, and Occludin, in the colon tissue of mice treated with IRN, indicative of an improved gut barrier function and potentially beneficial to skeletal health. Although the modulation of gut microbiota, SCFAs, and the integrity of the intestinal barrier may explain the curative potential of IRN against excessive bone loss, the precise regulations between gut microbiota and bone metabolism remain to be clarified. It is important to note that the OVX model, while simulating osteoporosis caused by estrogen deficiency, cannot fully recapitulate the multifactorial pathogenesis of human OP. To better assess the translational potential of IRN, future studies should employ complementary osteoporosis models, for example, aging or glucocorticoid‐induced models, to capture different aspects of human pathophysiology.

## 5 Conclusion

In summary, this study demonstrates the protective effects of IRN against OVX‐induced OP, primarily through modulation of inflammatory responses and oxidative stress, regulation of gut microbiota composition, SCFAs production, and protection of the intestinal barrier. This study provides preclinical evidence supporting the potential therapeutic value of IRN for osteoporosis and provides new insights into the development of natural products in the management of osteoporosis. Future research should focus on elucidating the molecular mechanisms involved to optimize the benefits of IRN in managing osteoporosis.

## Author Contributions

**Yitao Lei:** data curation (equal), investigation (equal), validation (equal), visualization (equal), writing – original draft (equal). **Xiao Zhao:** conceptualization (equal), investigation (equal), resources (equal), writing – review and editing (equal). **Shihui Liang:** data curation (equal), methodology (equal), supervision (equal), writing – review and editing (equal). **Yanming Liu:** data curation (equal), investigation (equal), methodology (equal), validation (equal), writing – review and editing (equal). **Yilin Zhou:** data curation (equal), methodology (equal), validation (equal), writing – review and editing (equal). **Yingtong Zhang:** investigation (equal), validation (equal), writing – review and editing (equal). **Shimei Li:** investigation (equal), validation (equal), writing – review and editing (equal). **Qunwei Dong:** funding acquisition (equal), resources (equal), supervision (equal), writing – review and editing (equal). **Ping Sun:** resources (equal), supervision (equal), writing – review and editing (equal).

## Conflicts of Interest

The authors declare no conflicts of interest.

## Data Availability

The data that support the findings of this study are available from the corresponding author upon reasonable request.
